# Blood donation during natural disasters – experience with COVID-19 and earthquakes in Croatia

**DOI:** 10.3325/cmj.2021.62.196

**Published:** 2021-04

**Authors:** Irena Jukić, Ana Hećimović, Tomislav Vuk

**Affiliations:** Croatian Institute of Transfusion Medicine *irena.jukic@hztm.hr*

The SARS-CoV-2 pandemic has decreased the blood component supply around the world, imposing numerous challenges on transfusion services. Croatia has not been an exemption, with a declining number of blood donors because of a limited population movement. The blood component supply in Croatia has also been affected by a variable consumption in hospitals, which had to be adjusted to epidemiological needs. The consumption was significantly lower in the beginning of spring and higher in the summer and early fall of 2020. 

Since the beginning of the pandemic, Croatia has been hit by several strong earthquakes, some of them devastating. On March 22, 2020, a 5.7-magnitude earthquake hit Zagreb, causing considerable material damage. About nine months later, on December 29, an even stronger (6.2-magnitude) earthquake struck the town of Petrinja, located about 50 km to the southeast. These two earthquakes corresponded respectively with the first and the second pandemic wave in Croatia. The Petrinja earthquake killed several people and injured many. Numerous people were left homeless. In the immediate aftermath of both earthquakes, cold weather and snow complicated the already difficult situation – a scenario that would seem unrealistic even in a movie. 

At the time of the Petrinja earthquake, the Croatian Institute of Transfusion Medicine (CITM) had a low blood component stock as a consequence of the pandemic. The Institute immediately sent a large supply of blood components to the hospital located near the epicenter. About two hours after the earthquake, people started coming to the CITM wishing to donate blood, their numbers increasing by the hour. Out of 336 people who wanted to donate blood that afternoon and evening, 246 made the donation. In comparison, the usual number of donations on workdays is 120, which means that the daily quota was exceeded by 205%. As many as 84 (34.1%) people were first-time donors. The next day, donors were waiting in front of the CITM from as early as 06:30 am (before the regular working hours, which start at 07:30). A long queue was formed in compliance with the pandemic protocols, stretching far into the street. Even though all available staff and equipment were mobilized, some people still had to wait for hours to give blood. The total number of blood donations that day was 475 (which is 395.8% more than the usual number), again with numerous first-time donors (207 or 43.6%). This number would have been even greater had we not asked the people arriving after 2 pm to return the following days or weeks to more evenly distribute the donation flow. This increased the donor turnout to the CITM and mobile donor sessions in the following days.

The number of donations in December 2020 was 4.6% higher than the number of donations in the same month of the previous year (*P* < 0.0001), despite the smaller total number of donors in 2020 due to the unfavorable epidemiological situation (Table 1). In December 2020, the number of female blood donors (*P* = 0.0016) and first-time donors (*P* < 0.0001) significantly increased compared with December 2019. The number of deferred donors (*P* = 0.045) also increased, which is a consequence of a large number of first-time donors, female donors (mostly deferred due to low hemoglobin), and the peak of the SARS-CoV-2 epidemic. Namely, not enough time had passed since COVID-19 recovery for some people to be able to donate blood safely. 

Similar results were obtained when comparing the data on the number of units collected during the last three days of 2019 and the last three days of 2020. We observed a significant increase in the number of female donors (*P* < 0.0001), who make around 16% of donors in Croatia. On December 30, ie, the day after the devastating earthquake, the female share reached as much as 32%. The number of first-time donors (*P* < 0.0001) and deferred donors also increased (*P* = 0.0045), which is unsurprising considering the circumstances and the motives. 

A higher turnout of blood donors continued into 2021, as confirmed by comparing the number of blood donations between December 29, 2019 and January 9, 2020 with the number of donations collected in the same 2020/2021 period (Table 1). It is important to highlight that, along with a higher turnout of regular and first-time donors (*P* < 0.0001), we observed more deferred donors (*P* < 0.0001), which could also be explained by the reasons stated above. During the investigated two-week period, both mobile donor sessions and CITM recorded significantly more potential blood donors compared with the regular period (172.3% more on average). The number of donations was on average higher by 138%, ranging from 30% to 395%. 

The aim of this report, which we are writing while the ground is still shaking, is to document not only the tragedy and sufferings, but also the kindness and solidarity of people who suppressed the fear of the pandemic to help their fellow citizens. We thank them for that. 

**Table 1:** The number of blood donations collected at the Croatian Institute of Transfusion Medicine in the investigated periods of 2019 and 2020

Time range	Number of donations (%)	P (X^2^)	M (%)	F (%)	P (X^2^)	Donations of first time donors (%)		Deferred donors (% of attended)	P (X^2^)
December	%/y								
2019	7912 (7.8)	<0.0001 (120.53)	6584 (83.2)	1329 (16.8)	0.0016 (9.99)	364 (4.6)	<0.0001 (74.77)	1675 (17.5)	0.045 (4.05)
2020	8279 (9.2)		6733 (81.3)	1546 (18.7)		651 (7.9)		1895 (18.6)	
December 29-December 31	%/December								
2019	498 (6.3)	<0.0001 (772.95)	419 (84.1)	79 (15.9)	<0.0001 (27.09)	14 (2.8)	<0.0001 (111.26)	99 (16.6)	0.0045 (8.06)
2020	1777 (21.5)		1291 (72.7)	486 (27.3)		424 (23.9)		498 (21.9)	
December 29-January 9	%/December +9 days								
2019/2020	2753 (27.1)	<0.0001 (545.15)	2428 (88.2)	325 (11.8)	<0.0001 (142.3)	91 (3.3)	<0.0001 (258.9)	425 (13.4)	<0.0001 (52.53)
2020/2021	4445 (42.5)		3418 (76.9)	1027 (23.1)		684 (15.4)		1079 (19.5)	

*test for difference in proportions.

